# An Analysis of Scotopic Microperimetry in Healthy Adults

**DOI:** 10.1167/tvst.14.6.18

**Published:** 2025-06-09

**Authors:** Daniel A. O. Adeyoju, Amandeep S. Josan, Laura J. Taylor, Robert E. MacLaren

**Affiliations:** 1 Oxford Eye Hospital, Oxford University Hospitals NHS Trust, Oxford, UK; 2 Nuffield Laboratory of Ophthalmology, Department of Clinical Neurosciences, University of Oxford, NIHR Oxford Biomedical Research Centre, Oxford, UK

**Keywords:** S-MAIA, scotopic microperimetry, dark-adapted fundus controlled perimetry, two-color perimetry

## Abstract

**Purpose:**

Scotopic microperimetry measures retinal sensitivity under very low light and may be useful in conditions characterized by nyctalopia, such as retinitis pigmentosa and age-related macular degeneration. The Scotopic Macular Integrity Assessment device enables two-color perimetry to isolate rod and cone responses. This study assesses the reliability, test–retest repeatability, and sensitivity in healthy participants aiming to establish normative values.

**Methods:**

Scotopic microperimetry was performed using cyan and red stimuli on a 37-point radial grid after dark adaptation on control participants with no history of eye disease and visual acuity of 0.1 logarithm of the minimum angle of resolution or better. Fixation stability, fixation losses, and identification of the rod-free zone were used as reliability metrics. A subset underwent repeat testing within 4 weeks.

**Results:**

Thirty-nine participants (19 male and 20 female), median age 24 years (interquartile range, 9.5 years) and 23 years (interquartile range, 9 years) for the right and left eyes, respectively, completed testing. Overall 77 eyes underwent scotopic testing, with 82% meeting reliability criteria. Mean cyan and red sensitivities were 19.9 ± 1.1 dB and 20.9 ± 1.2 dB in right eyes, and 20.1 ± 1.4 dB and 21.3 ± 1.4 dB in left eyes, respectively. Volumetric cyan and red sensitivities were 2868 ± 157 dB.deg^2^ and 3077 ± 176 dB.deg^2^ in the right eyes, respectively, and 2892 ± 205 dB.deg^2^ and 3126 ± 207 dB.deg^2^ in the left eyes, respectively. Mean sensitivity coefficients of repeatability (CoR) were ± 1.4 dB (cyan) and ± 2.1 dB (red) while pointwise coefficients of repeatability were ± 7.2 dB (95% confidence interval, 6.5–7.6 dB) for cyan and ± 7.9 dB (95% confidence interval, 7.3–8.4 dB) for red, with no significant differences between eyes or genders. Fixation stability assessed using the 95% bivariate contour ellipse area for cyan was 2.9 ± 5.9 deg^2^ and 2.3 ± 2.2 deg^2^ for the right and left eyes, respectively, and for red were 0.7 ± 0.6 deg^2^ and 0.9 ± 0.8 deg^2^ for the right and left eyes, respectively. Again, there were no significant differences between cyan and red tests (Friedman test, bivariate contour ellipse area 63%, *P* = 0.455; bivariate contour ellipse area 95%, *P* = 0.432).

**Conclusions:**

Scotopic microperimetry using the Scotopic Macular Integrity Assessment device was feasible and well-tolerated. Repeatability metrics demonstrated limitations in fine spatial mapping of scotopic retinal sensitivity.

**Translational Relevance:**

This study highlights potential areas for future improvements in scotopic microperimetry before its use as an outcome measure in clinical trials for retinal disease.

## Introduction

Microperimetry, also known as fundus-controlled perimetry, assesses central retinal function. It is being increasingly adopted as an outcome measure in clinical trials investigating potential novel therapies for inherited retinal diseases such as retinitis pigmentosa, Stargardt disease and age-related macular degeneration.^2^^,^^3^ Scotopic microperimetry is a modified version of standard mesopic microperimetry, assessing central retinal sensitivity at much lower light levels (background luminance of <0.001 cd/m^2^). Many eye diseases, such as age-related macular degeneration and retinitis pigmentosa, often manifest with nyctalopia, indicating a reduction in low-light visual function. Scotopic microperimetry, therefore, may provide a more sensitive functional measure of disease progression.^4^^–^^7^

The Scotopic Macular Integrity Assessment (S-MAIA; Centervue S.p.A., Padua, Italy) combines microperimetry testing with the concepts of two-color perimetry, presenting stimuli at two different wavelengths, thus allowing for preferential isolation of rod versus cone dominant responses at individual retinal locations.^8^ This allows for mapping of central retinal sensitivity by photoreceptor class, which is not possible with traditional global functional tests of rod and cone function, such as full-field stimulus testing or electroretinography. Given that the natural history of many ocular diseases involves early dysfunction in dark-adapted vision, it is pertinent to first understand scotopic microperimetric function in healthy eyes to establish normative ranges before investigating deviations from this in compromised eyes.

This study uses the S-MAIA device with the extended dynamic range of 0.0 to 36.0 dB, capable of two-color fundus-controlled perimetry with cyan (505 nm) and red (627 nm) stimuli. The use of scotopic microperimetry as an outcome measure in age-related macular degeneration is undergoing further clinical validation in large-scale trials, including MACUSTAR (Development of Novel Clinical Endpoints in Intermediate AMD)^9^ and ALSTAR2 (Alabama Study on Early Age-related Macular Degeneration 2)^10^ studies.

The standard output from the S-MAIA includes single loci point threshold sensitivities (often referred to as pointwise sensitivities), reported in decibels, is the mean sensitivity representing an average of all pointwise sensitivities. However, there are pros and cons to the use of both pointwise and mean sensitivity metrics. Pointwise sensitivities relay the most detailed information regarding retinal sensitivity, but are prone to high levels of variability. Mean sensitivity minimizes individual point variability, but also removes any spatial information from the testing grid. In addition, owing to the length of time taken to perform the test, scotopic microperimetry is often performed using testing grids with a nonuniform test point arrangement, such as radial testing grids, which have a greater density of test points either around the central fovea or in the parafoveal region. As such, any averaging of pointwise sensitivities becomes a weighted average, being heavily biased toward regions with a greater density of points. Another issue with mean sensitivity is due to a feature of microperimetry devices and their decibel treatment of nonseen stimuli. On a logarithmic scale, the dynamic range of perimeters scales from the brightest possible stimuli (defined as 0.0 dB for all devices) to the dimmest stimuli (36.0 dB in the case of MAIA). Hence, stimuli that are seen at the brightest possible level are assigned the value 0.0 dB. As such, loci that are not seen at the brightest level are arbitrarily assigned a sensitivity value of −1.0 dB. In patients with advanced ocular disease, who may have large regions with no detectable sensitivity, the mean sensitivity is composed of several pointwise sensitivities that have a value of −1.0 dB. This creates an issue with the mean sensitivity being weighted heavily toward zero decibels, creating an artificial floor effect.^11^^,^^12^ These issues can be overcome by adopting a volumetric sensitivity approach, representing total sensitivity with a three-dimensional hill of vision, where the volume sensitivity is given in either decibel-steradians (dB-Sr) or decibels-degrees squared (dB-deg^2^).^11^^,^^13^ Here, seen stimuli at the brightest level are assigned a small but nominal value of 0.001 dB and nonseen stimuli at the brightest level are reassigned to 0.0 dB. This approach removes the averaging toward zero issue, while the spatial element of calculating volume under a hill of vision simultaneously solves the spatial weighting issue in radial grid designs. Hill-of-vision models in mesopic microperimetry have been explored recently in normative datasets to find the best interpolation fitting function.^14^ Volumetric approaches have been applied previously with microperimetry for longitudinal assessment of patients with Stargardt disease^15^^,^^16^ and choroideremia.^17^ Although the usefulness of scotopic microperimetry has been explored in healthy controls,^18^ with no known ocular disease, the use of volumetric sensitivity indices to represent scotopic microperimetry remains to be explored.

In this study, we aimed to explore the performance of healthy participants during scotopic microperimetry testing using a S-MAIA device. We assess how reliably healthy participants can perform scotopic microperimetry by evaluating fixation losses and fixation stability metrics. We analyze the sensitivity data using volumetric hill of vision indices and seek to produce normative values for volumetric sensitivity to aid in sensitivity interpretation. Finally, we assess test–retest repeatability with both pointwise and volumetric measures.

## Methods

Participants were recruited as part of the Visual Function in Inherited Retinal Disease study (ISRCTN registration number: ISRCTN24016133, UK research ethics approval reference: 20/WM/0283).^12^ This study was conducted in accordance with the Declaration of Helsinki and all participants provided informed written consent. Thirty-nine participants were recruited from medical students, family members of patients, and colleagues. Inclusion criteria for the study were 18 to 85 years of age, with a visual acuity of 6/7.5 (0.1 logarithm of the minimum angle of resolution) or better. Exclusion criteria were a history of amblyopia or any known ophthalmic disease or surgery with long-lasting effects on visual function. The right eye was tested first for each participant, followed by the left eye. Testing was performed in a dedicated microperimetry testing room, which is a windowless room with all light sources omitted to ensure scotopic conditions (<1.0 lux), for dark adaptation, participants sat for 20 minutes in this darkened room before testing and were advised not to view mobile phones or smart watches.^19^

Two-color scotopic microperimetry was performed with the S-MAIA using the standard manufacturer's default 37-point radial testing grid and a fixation ring target with an intensity of 5.0 log units. The choice of grid is of great importance, being a compromise between resolution and length of time taken to complete. Owing to the difficulties in performing scotopic microperimetry and the need to perform the test twice using two different colored stimuli, the 37-point grid is often used in scotopic microperimetry whereas a 68-point grid is usually employed in mesopic microperimetry. Cyan (505 nm) stimuli testing was performed before red (627 nm) stimuli to minimize disrupting the dark-adapted state of rods. Testing was performed with no pharmacological pupil dilation.^20^ The basic design of the S-MAIA has been described previously.^21^ The testing grid comprises 3 concentric rings displaced from a central point at 3°, 5°, and 7°. A red ring with 1° diameter size was used as the fixation target. Stimuli were presented using an automated 4-2 staircase bracketing strategy to obtain the final threshold sensitivity. The minimum and maximum luminance capability within the S-MAIA for stimuli are 6.28 × 10^−^^5^ cd/m^2^ and 0.25 cd/m^2^, representing a dynamic range of 0.0 to 36.0 dB.^8^^,^^22^

False positives were evaluated by fixation losses, recorded as the percentage of positive responses to a stimulus presented at the centre of the optic nerve head (Heijl–Krakau method).^23^ Participants with fixation losses of greater than 20% were excluded from the final analysis. Fixation stability was evaluated using the bivariate contour ellipse area (BCEA), defined as an ellipsoid area which encompasses 63% (BCEA63) and 95% (BCEA95) of all fixation points, reported in degrees squared. Additionally, fixation stability can also be assessed by the percentage of fixation points recorded within a circle of radius 1° and radius 2° with respect to the preferred retinal locus, known as P1 and P2, respectively. By default within the microperimeter device, fixation is regarded as stable if more than 75% of the fixation points were located within P1; by contrast, fixation is regarded as unstable if fewer than 75% of fixation points were located within P2.^24^ Identification of the physiological rod-free zone in the central fovea was used as an additional indicator of response reliability in this study. Under scotopic conditions, it is assumed that cyan sensitivity at the central point should be significantly lower than any other pointwise sensitivity and should approach (or equal) 0.0 dB to be deemed a positive identification of the rod-free zone.

All examinations were carried out in the presence of a trained examiner. Full instructions were delivered to the participants before dark adaptation, with verbal encouragement during the test to maintain attentiveness. Participants were familiar with microperimetry as they had previously completed mesopic microperimetry testing, and so no formal training test was adopted. To assess repeatability, a subcohort underwent repeat testing in the right eye only within 4 weeks of the initial test. All subjects were invited to return and repeat testing was based on participant availability.

### Statistical Analysis

Statistical analysis was performed using SPSS (Ver 28.0, IBM, Armonk, NY) and R statistical programming language.^25^ Hill-of-vision volumetric analysis was performed using a custom written open source program that converts S-MAIA raw data files into a three-dimensional hill of vision representation of retinal sensitivity, with units dB-degrees^2^, described in detail previously by Josan et al.^11^ A paired *t* test was used to assess symmetry between right and left eye volumetric sensitivities. Spearman's correlation was used to assess change in volumetric sensitivity with age. Test-retest reliability was evaluated using the coefficient of repeatability (CoR), the range within which there is a 95% probability that two measurements on the same patient should lie, and by producing Bland–Altman plots^26^ for both pointwise sensitivity (using a linear mixed model framework) and volumetric sensitivity.

## Results

### Demographics

A total of 39 subjects were recruited between August 2021 and July 2022. These individuals underwent scotopic cyan and red microperimetry. All completed testing in the right eye and 38 completed testing in the left eye; one individual's left eye was excluded owing to a history of a retinal bleed. A subset of 16 completed repeat testing on the right eye only on a separate day within 4 weeks of the initial testing.

### Reliability

Individual eyes were excluded from further analysis if they showed more than 20% fixation losses in either the cyan or red tests. Six right eye tests, four left eye tests, and four right eye repeat tests were excluded on this basis, representing 18% of the total number of tests. The majority of remaining tests had 0% fixation losses with both cyan and red stimuli.

**Figure 1.**
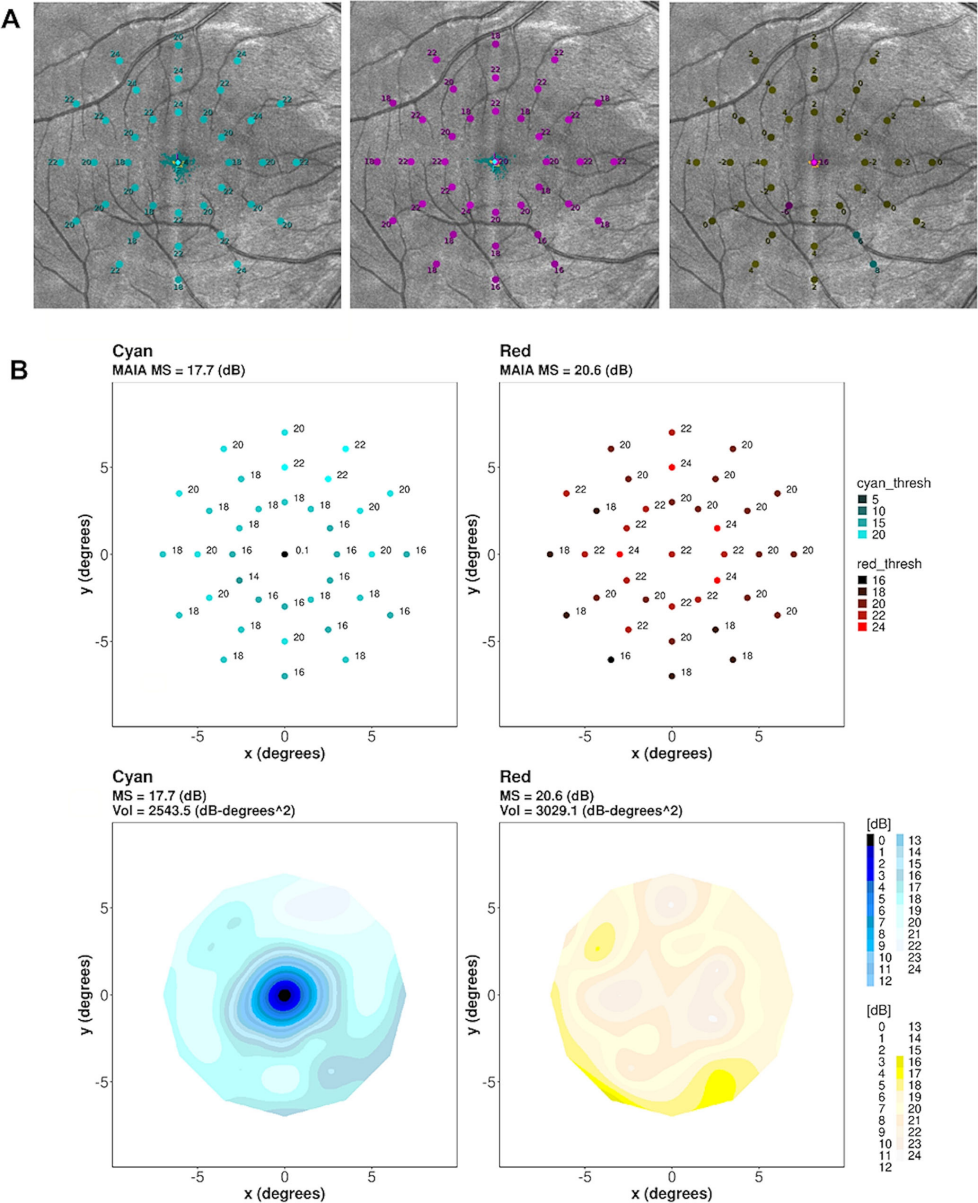
(**A**) An example scotopic test output, depicting cyan, red and cyan-red difference plot, superimposed on the fundus photography. The numeric value represents the measured threshold in decibels. (**B**) Custom program output where retinal sensitivity values and locations are transformed to a three-dimensional plot—the *X*–*Y* plane retains the position of the test points on the grid, and the *Z* axis is scotopic threshold sensitivity, forming a hill of vision via interpolation. The volume under this hill of vision measures the total amount of sensitivity in decibels degrees.^2^

Additional measures of test reliability were applied. P1 was greater than 75% in all but one test (Fig. 1), indicating that most participants had stable fixation throughout testing. One participant had reduced fixation during their first test, but showed stable fixation in all subsequent tests, showing a possible learning effect.

**Figure 2.**
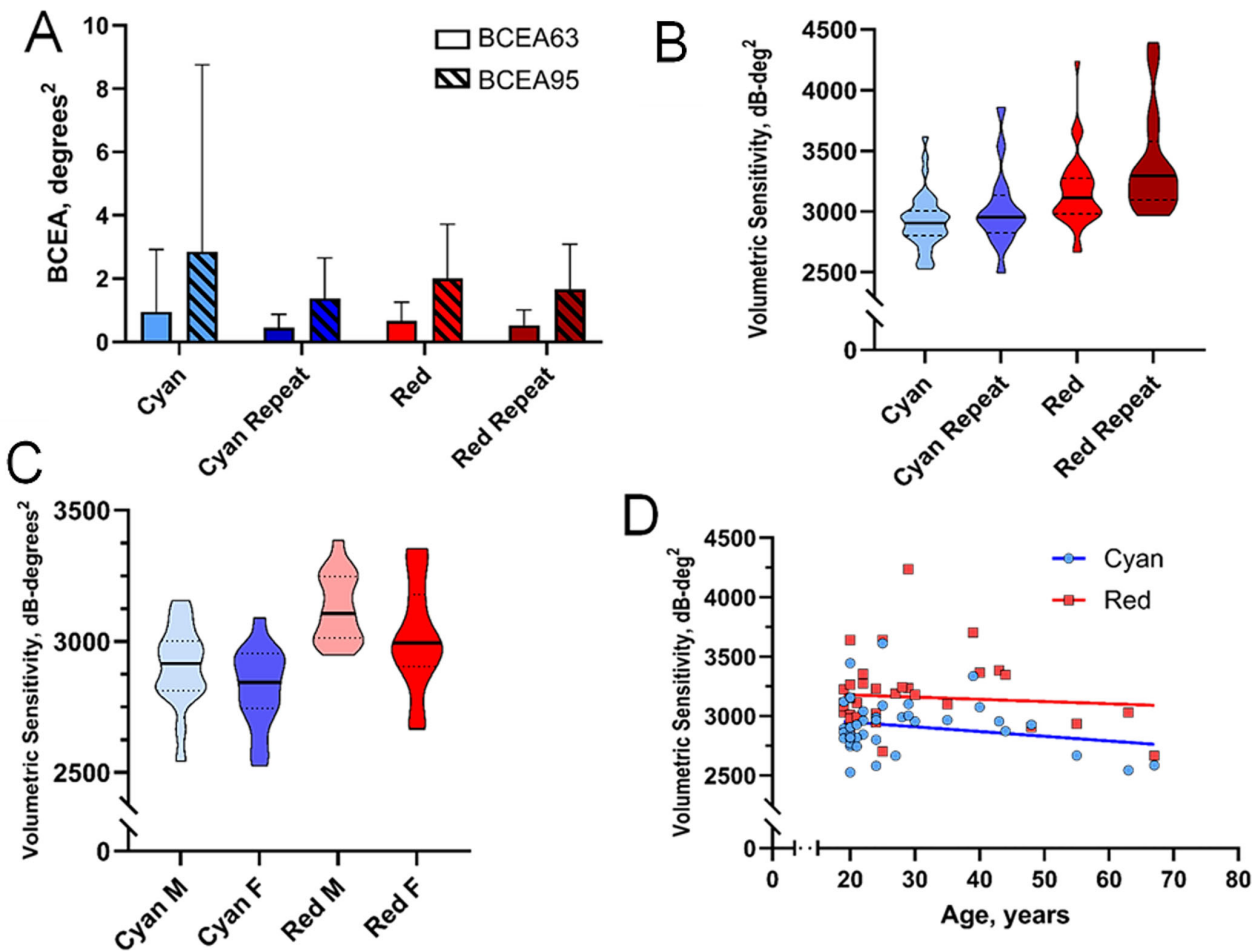
(**A**) Low 63% and 95% BCEA values indicating stable fixation. (**B**) Distribution of cyan and red volumetric sensitivity. (**C**) No significant difference in volumetric sensitivity between male (M) participants and female (F) participants with less than 20% fixation losses was seen (18 male; 15 female). (**D**). Volumetric sensitivity plotted against age of participants showed no significant relationship.

BCEA values were consistently low across the sub-cohorts indicating excellent fixation performance (Fig. 2A). There was no significant difference in BCEA between right and left eyes, between cyan and red tests or between the initial and repeat tests (Table 1) (Friedman test; BCEA63, *P* = 0.455; BCEA95, *P* = 0.432). The mean of the single central cyan point of the right eye, left eye and right eye repeats were between 5 and 6 dB. Central cyan sensitivity of more than 8 dB would be outside of the upper quartile of central cyan point sensitivity as reported in previous work^27^ and suggests poor rod-free zone mapping, suggesting greater false-positive responses and weaker reliability. This result was found in 18% of all eyes, and 14% of eyes after exclusion of those with poor fixation. Overall, there was no obvious trend in reliability metrics to suggest any learning or fatigue effects.

**Figure 3.**
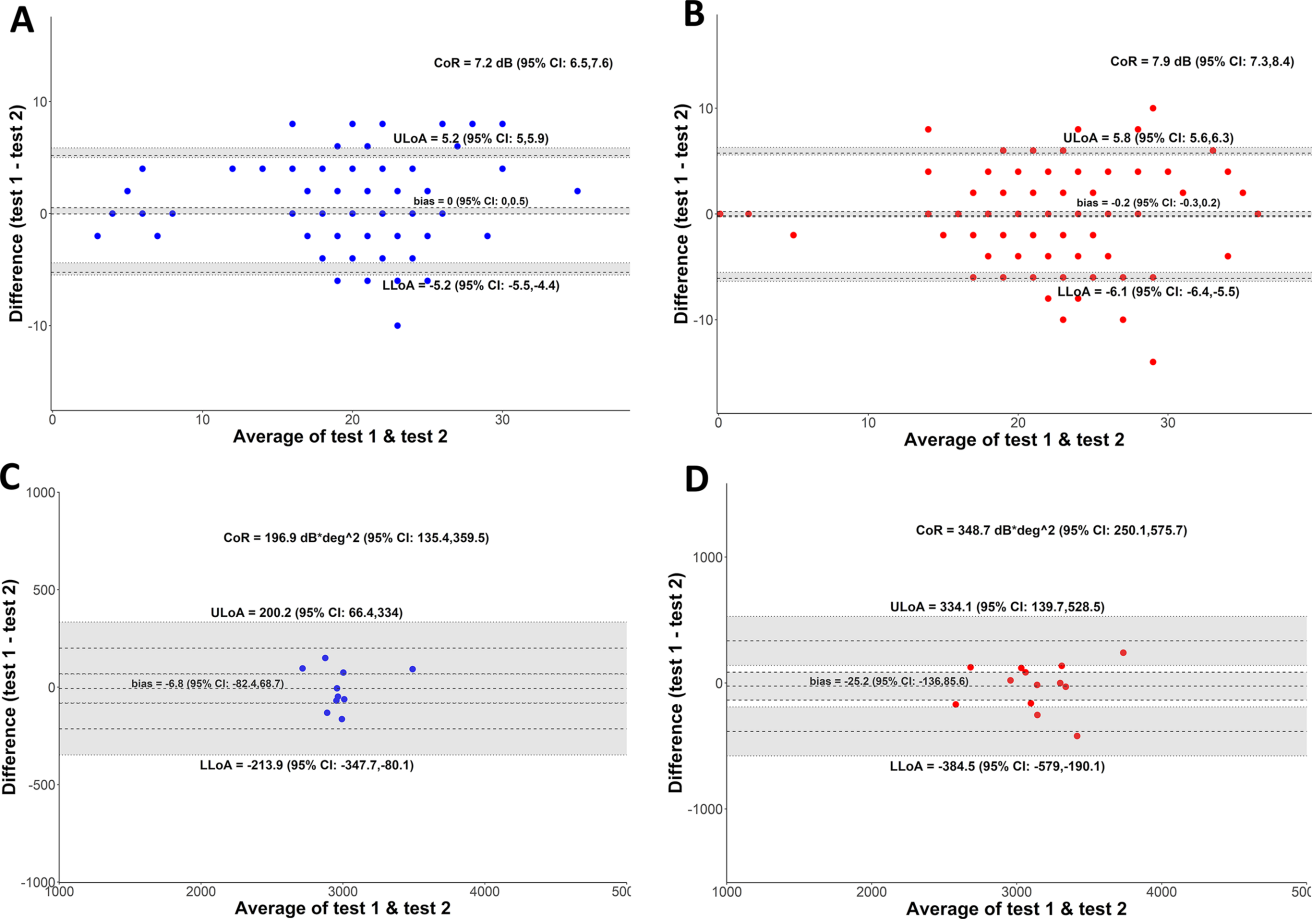
Bland–Altman plots. Pointwise test–retest repeatability for (**A**) cyan and (**B**) red. Volume of the hill-of-vision test–retest repeatability for (**C**) cyan and (**D**) red.

**Table 1.** Demographics and Reliability Indices

	Right Eye (*n* = 39)	Left Eye (*n* = 38)	Right Eye Repeat (*n* = 16)
Demographics			
Median age, years (IQR)	24 (9.5)	23^9^	26 (5.25)
Male:female	19:20	19:19	7:9
Reliability			
Participants with ≤20% fixation losses	33	34	12
Mean BCEA63 cyan, degrees^2^ ± SD	1.0 ± 2.0	0.8 ± 0.7	0.5 ± 0.4
Mean BCEA63 red, degrees^2^ ± SD	0.7 ± 0.6	0.9 ± 0.8	0.5 ± 0.5
Mean BCEA95 cyan, degrees^2^ ± SD	2.9 ± 5.9	2.3 ± 2.2	1.4 ± 1.3
Mean BCEA95 red, degrees^2^ ± SD	2.0 ± 1.7	2.8 ± 2.5	1.7 ± 1.4
Median cyan P1	99	98	99
Median red P1	98	95	99
Median cyan P2	100	100	100
Median red P2	100	100	100
Mean cyan central point ± SD	5.1 ± 4.1	5.9 ± 6.6	5.5 ± 3.8

IQR, interquartile range; SD, standard deviation.

These comprise a total of 186 tests across the cohort, as cyan and red testing are undertaken separately.

### Macular Sensitivity and Volumetric Analysis

In healthy participants, the mean cyan sensitivity was 20.0 ± 1.1 dB and 20.1 ± 1.4 dB in the right and left eyes, respectively. The mean red sensitivity was 20.9 ± 1.2 dB and 21.3 ± 1.4 dB in right and left eyes, respectively. The effective dynamic range was calculated as detailed by Wall et al.^28^ and defines the range of values the perimeter measures, which may be deemed clinically significant. The ceiling was 32.0 dB for cyan stimuli and 34.0 dB for red, defined as the value above which less than 0.5% of the values fall in normal eyes. This finding suggests the upper bound of dynamic range of 36.0 dB is sufficient in both cyan and red cases. The floor was not able be determined in normal eyes owing to the absence of abnormal scotomata.

For volumetric sensitivity, the mean cyan volumetric sensitivity was 2868 ± 157 dB.deg^2^ and 2892 ± 205 dB.deg^2^ in the right and left eyes, respectively. The mean red volumetric sensitivity was 3077 ± 176 dB.deg^2^ and 3126 ± 207 dB.deg^2^ in the right and left eyes, respectively. There was no significant difference in mean volumetric sensitivity between right and left eyes (paired *t* test: cyan, *P* = 0.452; red, *P* = 0.569; cyan–red, *P* = 0.333). Owing to the interocular symmetry, we present the right eye only in the following analysis (Fig. 2). Mean volumetric sensitivity in female participants was 2824 dB.deg^2^ for cyan and 3016 dB.deg^2^ for red and in males, 2904 dB.deg^2^ for cyan and 3127 dB.deg^2^ for red. There was no significant difference in volumetric sensitivity in the right eye between female and male participants with either cyan or red stimuli (*t* test: cyan, *P* = 0.149; red, *P* = 0.073) (Fig. 2C). There was also no significant correlation between age and volumetric sensitivity in the right eye (Spearman's correlation: cyan, ρ = −0.007, *P* = 0.971; red, ρ = −0.002, *P* = 0.993) (Fig. 2D).

### Repeatability

Twelve participants successfully completed repeat testing on the right eye only, after the exclusion of four participants with greater than 20% fixation losses. This produced a total of 444 repeated test loci for both cyan and red stimuli, allowing for the calculation of the CoR (Table 2).

**Table 2.** Mean Threshold and Volumetric Sensitivity in Test 1 and Test 2 Among Participants Who Completed Duplicate Testing (*n* = 12)

	Cyan Pointwise [95% CI] (dB)	Red Pointwise [95% CI] (dB)	Cyan MS (dB)	Red MS [95% CI] (dB)	Cyan Vol [95% CI] (dB.deg^2^)	Red Vol [95% CI] (dB.deg^2^)
Mean of test 1	–	–	20.1	21.4	2883	3144
Mean of test 2	–	–	20.9	22.1	3012	3257
Bias	0	−0.2	0	−0.2	−7	−25
CoR	±7.2 [6.5–7.6]	±7.9 [7.3–8.4]	±1.4 [1.0–2.6]	±2.1 [1.5–3.5]	±197 [135–360]	±349 [250–576]

CI, confidence interval.

Bias and CoR for pointwise, mean sensitivity (MS) and volumetric sensitivity (Vol).

The pointwise CoR, accounting for repeated measures and evaluated using Bland-Altman plots (Fig. 3), was ± 7.2 dB (95% confidence interval, 6.5–7.6) with cyan stimuli, ± 7.9 dB (95% confidence interval, 7.3–8.4) with red stimuli. This finding suggests that, on a pointwise level, a difference of more than approximately 8 dB is required to identify a clinically meaningful difference. The mean sensitivity in test two was 0.7 dB higher than test one for both cyan and red stimuli. Mean volumetric sensitivity was greater in test two by 128 dB.deg^2^ for cyan and 114 dB.deg^2^ for red stimuli. The CoR for both mean and volume sensitivity was higher with red than cyan (Table 2); however, none of these differences attained statistical significance.

## Discussion

This study demonstrates the application of volumetric analyses in scotopic central retinal sensitivity using the S-MAIA device with the extended dynamic range, providing a normative dataset for mean and volumetric sensitivity. The reliability of the dataset was ensured across multiple measures, including fixation losses, fixation stability, and the identification of a physiological rod-free zone. Assessment of scotopic function is more logistically complex to undertake and time consuming, in comparison with other forms of microperimetry, owing to the requirement for full dark adaptation before testing, followed by a challenging examination. Viewing threshold stimuli in complete darkness requires good concentration and lacks real-world generalizability.

Most participants were able to perform microperimetry reliably with 82% satisfying the inclusion criteria. Scotopic microperimetry testing is difficult and tiring for the participant. We believe the high rate of exclusions reflects the difficulty of the test. On the assumption that an examination is deemed reliable if it falls within the 95% confidence interval in a control group, our dataset indicates that in healthy participants, a BCEA95 of approximately 5 deg^2^ or less may be considered a stable fixation. BCEA values were consistent with previous measures of fixation stability on radial testing grids under scotopic conditions^18^ and mesopic conditions.^29^^,^^30^ P1 and P2 act as complementary measures of fixation stability and only one test in this study was considered relatively unstable for only meeting one of these criteria. Fixation stability is an important consideration in deciding on the reliability of any given examination. Previous work has found that owing to the limitations of the 25-Hz refresh rate of the scanning laser ophthalmoscope used to perform retinal tracking, a good fixation performance is required to avoid undetected stimulus placement errors owing to saccadic eye movements overwhelming the tracking capabilities.^31^ This is likely to be the source of high variability found at the borders of deep scotomas.^32^

Owing to the physiological absence of rod photoreceptors in the central fovea, cyan sensitivity at this location should have a significantly reduced or absent threshold value in comparison with the paracentral test loci. Therefore, positive identification of the rod-free zone acts as an additional measure of test reliability (incorporating both fixation stability and false positive testing). This assumes accurate positioning of the test grid and correspondence between the anatomical and physiological fovea. There are limitations to this approach: the 37-point radial default grid used in this study includes only a single central test point. Improving the validity of this approach may involve adapting the testing algorithm to include multiple test loci in the central fovea within the rod-free zone. Alternatively, introducing formal false-positive catch trials, following strategies used in static perimetry,^33^ may improve reliability assessment in microperimetry. Both options, however, would increase testing times significantly in an already long and difficult testing procedure.

A standard output of the device is the cyan minus red difference. The S-MAIA has calibrated the decibel values to account for greater retinal sensitivity toward cyan. Hence, loci with a negative cyan minus red value would theoretically indicate a cone dominant region, loci with a positive value would indicate a rod dominant region and those with zero value would indicate either healthy retina or equal rod/cone dysfunction. Whilst appealing, there is a great deal of potential for misinterpretation. For example, in the most extreme case of complete rod dysfunction in a given region, a cyan stimulus is unlikely to elicit no response at the brightest level (0.0 dB), but rather will elicit a response from any healthy cones that are present. This creates ambiguity in interpreting with confidence whether a region is truly rod or cone dominant. Additionally, when the S-MAIA reports mean difference, it does not account for the central rod-free zone, which skews toward a negative cyan–red difference. For this reason, we do not consider cyan minus red to be a viable metric and have not considered it in detail in this work.

Pfau et al^18^ (2017) investigated scotopic microperimetry in a similar cohort of participants with no visual impairment, however, this study only had a dynamic range of 0.0 to 20.0 dB (stimulus luminance range, 0.0025–0.25 cd/m^2^) and many participants experienced threshold ceiling effects. In this study, with the increased dynamic range (0.0–36.0 dB) we demonstrate the upper limit of effective dynamic range to be 32.0 dB for cyan and 34.0 dB for red stimuli. This finding suggests that the S-MAIA with extended dynamic range is sufficient in removing the ceiling effect relevant for healthy retinas.

In a recent study by the authors involving scotopic microperimetry in a cohort of choroideremia patients, repeatability for scotopic pointwise CoR was ±15.5 dB and ±12.4 dB, with a scotopic mean sensitivity CoR of ±3.3 dB and ±1.4 dB for cyan and red stimuli, respectively.^27^ In a similar study using mesopic microperimetry with patients with *RPGR*-related retinitis pigmentosa,^12^ it was reported that CoR for pointwise sensitivity were ±9.5 dB and ±9.3 dB, in the right and left eyes, respectively and the mean sensitivity CoR for the right and left eyes was ±0.7 dB and ±1.3 dB. Comparatively, in this study of healthy eyes, a difference exceeding approximately 8.0 dB on a pointwise level is necessary to identify meaningful changes. Our findings demonstrate a high pointwise CoR, in contrast with previous reports of the CoR for pointwise sensitivity in healthy controls.^18^^,^^34^ However, the CoR for mean sensitivity remains similar to previous studies.^35^^,^^36^ This discrepancy may be in a significant part owing to the underestimation of CoR where data are not nested in a linear mixed effect model framework. These findings may also suggest that repeatability under scotopic conditions is less robust than that seen in mesopic microperimetry, demonstrated by a CoR of 0.7 dB for mean sensitivity reported by Higgins et al.^36^ The relationship between stimulus location and threshold sensitivity/repeatability was not explored here. This has been previously explored by Welker et al.^35^ and Pfau et al.,^37^ who present normative data for scotopic microperimetry assessed using the MAIA device. They demonstrate there is little variation in sensitivity or repeatability at greater eccentricities under scotopic conditions.

This study has several limitations. First, there is a skew in the age of the study population toward patients aged 20 to 40 years. As a result, any trends in scotopic sensitivity or reliability in older participants may not have been captured adequately. Furthermore, there was no formal ocular assessment before recruitment, exclusion of participants with known eye disease, or a history of eye surgery was based on participants' reports. Therefore, participants may have been included who had unknown eye disease; however, because the cohort of participants was young, it is unlikely they would have had significant age-related ocular changes, such as cataracts or age-related macular degeneration. Pupil dilation was not performed. Previously, pupil dilation was shown to have no effect on mesopic microperimetry testing, provided patients had a minimum pupil diameter of 2.5 mm.^20^ Because, in scotopic testing conditions are darker this factor encourages natural pupil dilation, another study showed no clinically significant differences in scotopic perimetry sensitivity, using a modified Octopus 900 perimeter (Haag-Streit, Köniz, Switzerland), with and without pupil dilation.^34^ Therefore, pharmacological pupil dilation is unlikely to have a significant impact on the results. In addition, owing to the MAIA's autofocusing capabilities, spectacle correction is not required for testing, therefore refractive errors were not collected as part of this study and it was assumed participants fell within the MAIA's corrective range (−15.00 to +10.00 DS). The consistent use of the right eye for the repeatability testing was a further limitation. Randomizing the eye to be repeated would have been a more robust repeat testing regime. However, in a healthy control population such as this, we would not expect any significant changes to these results between eyes. Furthermore, the repeatability analyses are limited by a small number of repeat tests, repeatability using more tests would yield a greater degree of confidence in the resulting CoR values; however, it was not possible to ask patients to return on many occasions.

## Conclusions

In this study, we systematically assessed retinal sensitivity under scotopic conditions in healthy eyes using the S-MAIA device. Scotopic testing was feasible and well-tolerated in most healthy participants, demonstrating the potential usefulness of this method in clinical and research settings. A BCEA95 of approximately 5 deg^2^ or less indicates stable fixation. Repeat testing did not suggest any significant learning effects. Looking forward, future devices capable of scotopic microperimetry may enable more refined structure–function analyses in a range of retinal diseases and hold promise as an outcome measure in clinical trials. However, significant improvements in repeatability are essential before the method can be reliably applied in such settings.
